# A retrospective study on the trends in surgical aortic valve replacement outcomes in the post‐transcatheter aortic valve replacement era

**DOI:** 10.1002/hsr2.660

**Published:** 2022-05-22

**Authors:** Johnny Chahine, Zeina Jedeon, Jacob Fiocchi, Andrew Shaffer, Ryan Knoper, Ranjit John, Demetris Yannopoulos, Ganesh Raveendran, Sergey Gurevich

**Affiliations:** ^1^ Department of Cardiovascular Disease University of Minnesota Medical School Minneapolis Minnesota USA; ^2^ Department of Internal Medicine University of Minnesota Medical School Minneapolis Minnesota USA; ^3^ Department of Cardiothoracic Surgery University of Minnesota Medical School Minneapolis Minnesota USA

**Keywords:** aortic stenosis, outcomes, surgical aortic valve replacement, transcatheter aortic valve replacement

## Abstract

**Background and Aims:**

Transcatheter aortic valve replacement (TAVR) is the mainstay of treatment of inoperable and severe high‐risk aortic stenosis and is noninferior to surgical aortic valve replacement (SAVR) for low‐risk and intermediate‐risk patients as well. We aim to compare the valve size, area, and transaortic mean gradients in SAVR patients before and after the implementation of TAVR since being approved by the Food and Drug Administration  in 2011.

**Methods:**

Patients who underwent a bioprosthetic SAVR placement were divided into two groups based on the date of procedure: the early pre‐TAVR implementation group (years 2011–2012) and the contemporary post‐TAVR group (years 2019–2020). The primary endpoint was the mean gradient across the aortic valve within 16 months of surgery. The secondary endpoints included the difference in valve size and various aortic valve echocardiographic variables.

**Results:**

One hundred and thirty patients had their valves replaced in the years 2011–2012 and 134 in the years 2019–2020. The early group had a significantly higher mean gradient (median of 13 mmHg [interquartile range, IQR: 9.3–18] vs. 10 mmHg [IQR: 7.5–13.1], *p* = 0.001) and a smaller median effective orifice area index (0.8 cm^2^/m^2^ [IQR: 0.6–1] vs. 1.1 cm^2^/m^2^ [IQR: 0.8–1.3], *p* < 0.001). The median valve size was significantly smaller in the early group (median of 21 mm [IQR: 21–23] vs. 23 mm [IQR: 22.5–25], *p* < 0.001).

**Conclusion:**

In the contemporary era, surgical patients receive larger valves which translates into lower mean gradients, larger valve area, and lower rates of patient‐prosthesis mismatch than in previous years before the routine introduction of TAVR.

## 1 INTRODUCTION

Transcatheter aortic valve replacement (TAVR) is the mainstay of treatment of inoperable and severe high‐risk aortic stenosis (AS).^1^ It has been shown to be noninferior to surgical aortic valve replacement (SAVR) for low‐risk and intermediate‐risk patients as well.^2^ The success of TAVR in randomized controlled trials as well as in national and international registries has led to an increase in TAVR given better safety profile and postoperative recovery due to the less invasive approach.^3^ However, SAVR remains the standard of care for younger patients given the lack of data on TAVR durability long term as compared to surgical mechanical and even bioprosthetic valves.^4^

The hemodynamic profile of surgical bioprosthetic valves has always been inferior to the TAVR as shown in the PARTNER trials. The TAVR implants have consistently demonstrated lower mean gradients and greater valve area. While this was common in the inoperable, high‐risk, and intermediate‐risk trials, the last series of low‐risk randomized controlled trials displayed a trend toward lower gradients and areas even in the SAVR cohort. In this study, we aim to study and compare the size, valve area, and transaortic mean gradient in SAVR patients before and after the implementation of TAVR in treating AS since being approved by the Food and Drug Administration (FDA) in 2011.

## 2 METHODS

This is a single‐center retrospective study performed at the University of Minnesota Medical Center (UMMC). UMMC is a quaternary, 800‐bed hospital in Minneapolis, MN, and adopted the TAVR immediately after its approval by the FDA in 2011. Between 2012 and 2020, over 700 TAVR procedures have been performed along with several other structural procedures such as MitraClip, Watchman, and so forth. This study was approved by the University of Minnesota Institutional Review Board. Informed consent was waived given the retrospective nature of the study and the use of deidentified data in this manuscript.

A total of 264 patients were included in the study and retrospectively analyzed. Patients undergoing a bioprosthetic SAVR placement were included in this study. Excluded patients were those who had a mechanical valve placed, those with prior history of aortic valve replacement, those who had their valve replaced outside of the University of Minnesota Medical Center, and those with no transthoracic echocardiography follow‐up available within 16 months of valve implantation. Patients were further divided into two groups based on the date of procedure: the early pre‐TAVR implementation group (years 2011–2012) and the contemporary post‐TAVR group (years 2019–2020).

The primary endpoint was the mean gradient across the aortic valve within 16 months of surgery. The secondary endpoints included the size of the bioprosthetic aortic valve implanted, other aortic valve echocardiographic variables (peak velocity, valve area, and velocity ratio) as well as left ventricular ejection fraction within 16 months of surgery, 30‐day mortality, 1‐year mortality, need for TAVR after surgery, need for a permanent pacemaker within 30 days of the surgery, a new need for dialysis within 30 days of the surgery, and the occurrence of cerebrovascular events within 30 days of the surgery. Valve data were obtained from follow‐up echocardiographic data.

Baseline characteristics were collected from the electronic medical records: age, gender, body mass index, diabetes mellitus, chronic obstructive pulmonary disease (COOPD), hypertension, chronic kidney disease, smoking history, alcohol use, intravenous drug use, history of stroke or transient ischemic event, myocardial infarction, history of coronary artery bypass graft (CABG) or percutaneous coronary intervention, peripheral vascular disease, left ventricular ejection fraction before surgery, endocarditis as the indication for surgery, and the need for CABG or aortic root replacement during the procedure. Variables related to the endpoints above were also collected. The date of death was determined from the electronic medical record system and using an online obituary website legacy. com.

Continuous variables were stated as medians and interquartile ranges (IQR), and categorical variables as percentages. The *k*‐sample median test was used to assess continuous variables, and *χ* ^2^​​ and Fisher exact tests were utilized to assess categorical variables. Statistical analysis was executed using SPSS version 25.0 (IBM). A *p* < 0.05 was considered to be statistically significant.

## 3 RESULTS

**Figure 1:**
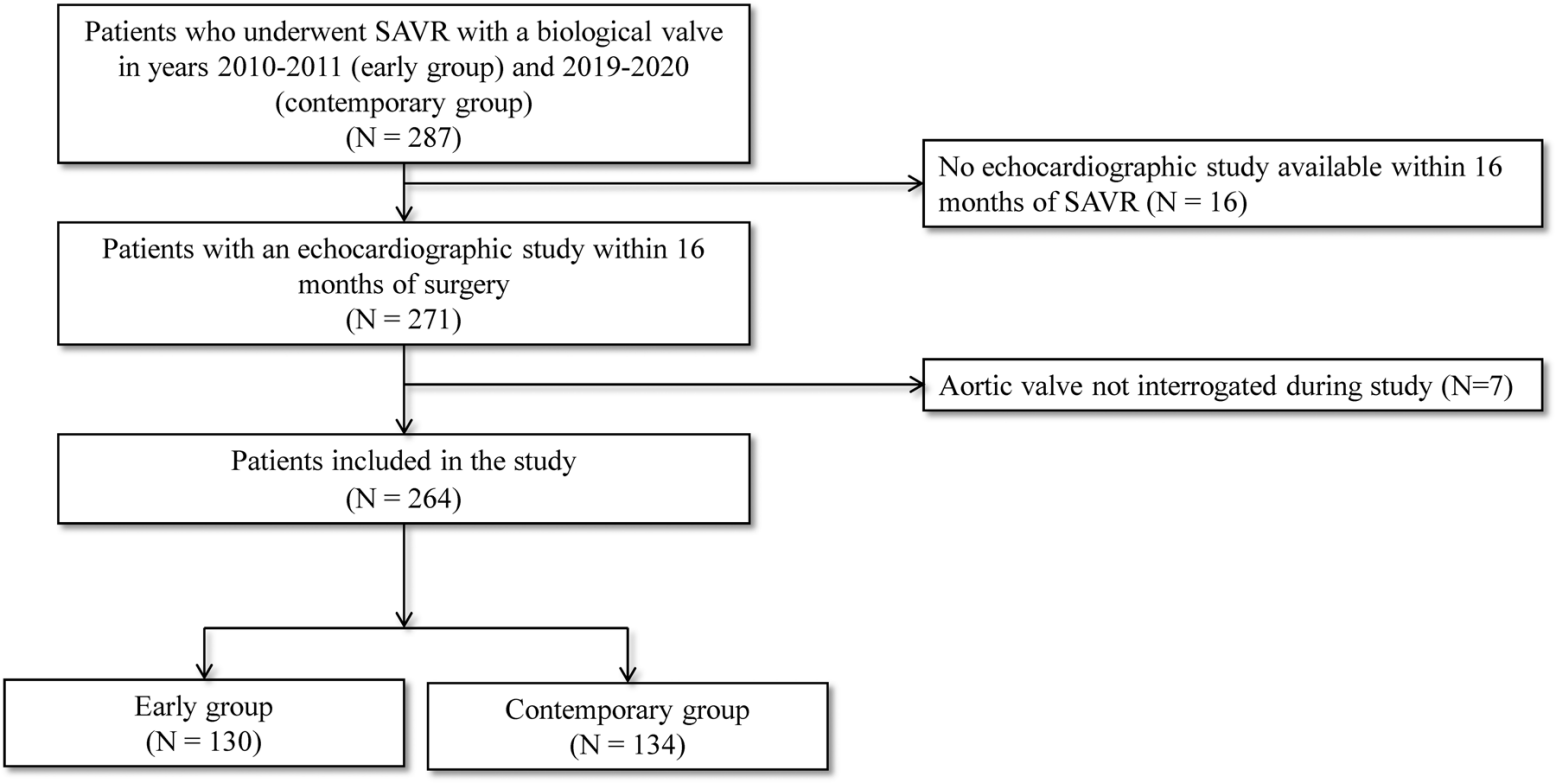
Study flow chart. Flow chart showing the selection process of the study population. Out of 287 patients who had SAVR in years 2010–2011 and 2019–2020, 23 patients were excluded based on unavailable or incomplete echocardiographic studies. Two hundred and sixty‐four patients were included, 130 patients had their valve replaced in years 2011–2012 (early group) and 134 in years 2019–2020 (contemporary group). SAVR, surgical aortic valve replacement.

Two hundred and sixty‐four patients were included in the study: 130 patients had their valve replaced in years 2011–2012 (early group) and 134 in years 2019–2020 (contemporary group; Figure 1). Patients in the early group were older (median age: 74 [IQR: 65.8–2] vs. 67.5 [IQR: 59.8–73], *p* = 0.001), more likely males (*n* = 76, 58.5% vs. *n* = 99, 73.9%, *p* = 0.008), had higher tobacco use (*n* = 75, 57.7% vs. *n* = 61, 45.5%, *p* = 0.048), more likely to have COPD (*n* = 42, 32.3% vs. *n* = 20, 14.9%, *p* = 0.001), hypertension (*n* = 122, 93.8% vs. *n* = 115, 85.8%, *p* = 0.03), chronic kidney disease (*n* = 67, 51.5% vs. *n* = 49, 36.6%, *p* = 0.01), and a previous cerebral ischemic event (*n* = 42, 32.3% vs. *n* = 23, 17.2%, *p* = 0.004). In the contemporary group, patients were more likely to use intravenous drugs (*n* = 0, 0% vs. *n* = 9, 6.7%, *p* = 0.003) and to have endocarditis as the indication for surgery (*n* = 4, 3.1% vs. *n* = 21, 15.7%, *p* < 0.001). Replacement of the aortic root was more prevalent in the contemporary group (*n* = 43, 32.1% vs. *n* = 19, 14.6%, *p* = 0.001; Table 1).

**Table 1:** Baseline characteristics

	Total	Years 2011‐2012	Years 2019‐2020	*p*
	*N* = 264	*N* = 130	*N* = 134	
Age (years), median (IQR)	70 (63–78)	74 (65.8–82)	67.5 (59.8–73)	0.001
Male, *n* (%)	175 (66.3)	76 (58.5)	99 (73.9)	0.008
Body mass index (kg/m^2^), median (IQR)	28.3 (25.1**–**32.4)	28 (25.5**‐**32)	28.7 (24.5**–**32.9)	0.5
DM, *n* (%)	98 (37.1)	51 (39.2)	47 (35.1)	0.49
COPD, *n* (%)	62 (23.5)	42 (32.3)	20 (14.9)	0.001
HTN, *n* (%)	237 (89.8)	122 (93.8)	115 (85.8)	0.03
ESRD, *n* (%)	6 (2.3)	3 (2.3)	3 (2.2)	1
Smoking history, *n* (%)	136 (51.5)	75 (57.7)	61 (45.5)	0.048
Alcohol use, *n* (%)	151 (57.2)	79 (60.8)	72 (53.7)	0.25
Intravenous drug use, *n* (%)	9 (3.4)	0(0)	9 (6.7)	0.003
Endocarditis as the indication for AVR, *n *(%)	25 (9.5)	4 (3.1)	21 (15.7)	<0.001
Previous stroke or TIA, *n* (%)	65 (24.6)	42 (32.3)	23 (17.2)	0.004
Myocardial infarction, *n* (%)	73 (27.7)	37 (28.5)	36 (26.9)	0.77
Previous CABG, *n* (%)	87 (33)	48 (36.9)	39 (29.1)	0.18
Previous PCI, *n* (%)	13 (4.9)	5 (3.8)	8 (6)	0.43
Peripheral vascular disease, *n* (%)	175 (66.3)	90 (69.2)	85 (63.4)	0.32
LVEF (%), median (IQR)	57.7 (47.5–65.1)	58.4 (47.5**–**66.7)	57.5 (47.4**‐**64)	0.27
CABG done during procedure, *n* (%)	87 (33)	46 (35.4)	41 (30.6)	0.41
Aortic root replacement during procedure, *n* (%)	62 (23.5	19 (14.6)	43 (32.1)	0.001

Abbreviations: AVR, aortic valve replacement; CABG, coronary artery bypass graft; COPD, chronic obstructive pulmonary disease; DM, diabetes mellitus; ESRD, end‐stage renal disease; HTN, hypertension, LVEF, left ventricular ejection fraction; PCI, percutaneous coronary intervention; TIA, transient ischemic attack.

In all, 26.9% of patients (*n* = 71) had an Inspiris Resilia valve implanted, 18.6% (*n* = 49) had a Carpentier‐Edwards Perimount Magna Ease valve, 14.8% (*n* = 39) had a Mitroflow pericardial heart valve, and 14.4% (*n* = 38) had a St Jude Medical Trifecta aortic valve.

**Figure 2:**
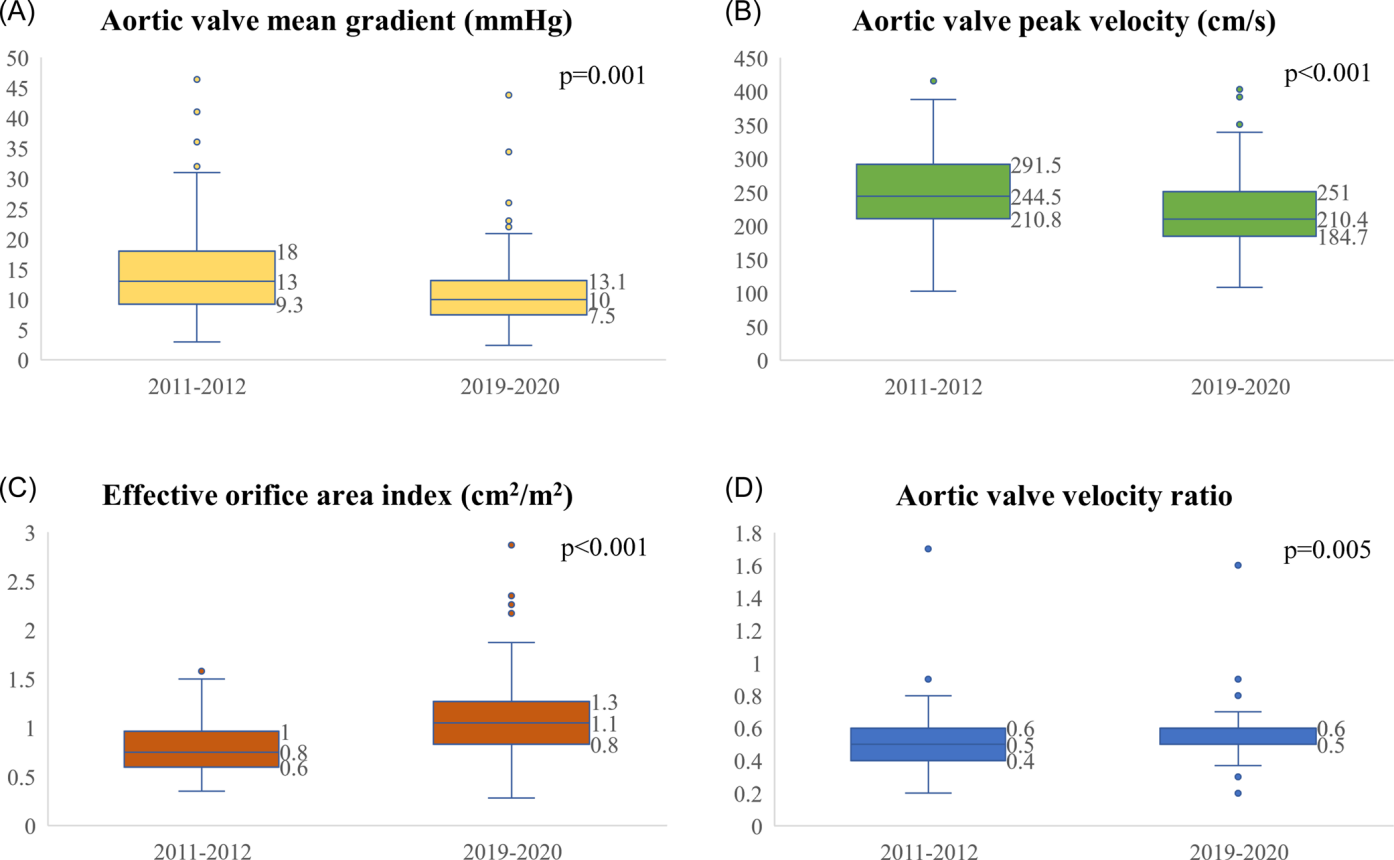
Echocardiographic variables comparison between the study groups. Boxplots comparing several echocardiographic variables between patients who had a bioprosthetic SAVR procedure in 2011–2012 (early group, pre‐TAVR implementation) and those who had the procedure in 2019–2020 (contemporary group, post‐TAVR implementation). The early group had a significantly higher mean gradient (median of 13 mmHg [IQR: 9.3–18] vs. 10 mmHg [IQR: 7.5–13.1], *p* = 0.001) (A), a higher median peak aortic valve velocity (244.5 cm/s [IQR: 210.8–291.5] vs. 210.4 cm/s [IQR: 184.7–251], *p* < 0.001) (B), a smaller median effective orifice area index (0.8 cm^2^/m^2^ [IQR; 0.6–1] vs. 1.1 cm^2^/m^2^ [IQR: 0.8–1.3], *p* < 0.001) (C), and a smaller median aortic valve velocity ratio (0.48 [IQR : 0.39–0.56] vs. 0.56 [IQR: 0.46–0.64], *p* = 0.005) (D). SAVR, surgical aortic valve replacement; TAVR, transcutaneous aortic valve replacement.

The mean gradient in the early group was significantly higher within 16 months of valve implantation with a median of 13 mmHg (IQR: 9.3–18) versus 10 (IQR: 7.5–13.1) in the contemporary group (*p* = 0.001; Figure 2A). The rate of mean pressure gradients above 20 was also higher in the early group (*n* = 21, 16.2% vs. *n* = 10, 7.5%, *p* = 0.03). The early group had a higher median peak aortic valve velocity (244.5 cm/s [IQR: 210.8–291.5] vs. 210.4 cm/s [IQR: 184.7–251], *p* < 0.001; Figure 2B), a smaller median effective orifice area (1.5 cm^2^ [IQR 1.2–1.8] vs. 2 cm^2^ [IQR 1.6–2.5], *p* < 0.001; Figure 2C), and a smaller median aortic valve velocity ratio (0.48 [IQR: 0.39–0.56] vs. 0.56 [IQR: 0.46–0.64], *p* = 0.005; Figure 2D and Table 2). The rate of patients with an effective orifice area index below 0.75 cm^2^/m^2^ was significantly higher in the early group suggestive of higher rates of patient‐prosthesis mismatch (*n* = 58 [49.6%] vs. *n* = 24 [20.2%], *p* < 0.001).

**Table 2:** Study outcomes

	Total	Years 2011–2012	Years 2019–2020	*p*
	*N* = 264	*N* = 130	*N* = 134	
LVEF after the surgery (%), median (IQR)	60.0 (47.6–65.0)	60.0 (47.5–65.0)	60.0 (48.9–65.0)	0.23
Stroke/TIA within 30 days of SAVR, *n* (%)	17 (6.4)	6 (4.6)	11 (8.2)	0.23
Need for permanent pacemaker within 30 days of SAVR, *n* (%)	12 (4.5)	5 (3.8)	7 (5.2)	0.56
Need for TAVR post SAVR, *n* (%)	1 (0.4)	1 (0.8)	0 (0)	0.49
New need for dialysis within 30 days of SAVR, *n* (%)	11 (4.2)	4 (3.1)	7 (5.2)	0.38
30‐day mortality, *n* (%)	5 (1.9)	2 (1.5)	3 (2.2)	1
1‐year mortality, *n* (%)	20 (7.6)	10 (7.7)	10 (7.5)	0.94
Size of valve (mm), median (IQR)	23.0 (21.0**–**25.0)	21.0 (21.0–23.0)	23.0 (22.5**–**25.0)	<0.001
Aortic valve mean gradient after the surgery (mmHg) (median, IQR)	11.0 (8.0–15.5)	13.0 (9.3–18.0)	10.0 (7.5–13.1)	0.001
Mean pressure gradient above 20 mmHg, *n* (%)	31 (11.7)	21 (16.2)	10 (7.5)	0.03
Mean pressure gradient above 30 mmHg, *n* (%)	9 (3.4)	7 (5.4)	2 (1.5)	0.01
Aortic valve peak velocity (cm/s), median (IQR)	230.0 (193.2–264.0)	244.5 (210.8–291.5)	210.4 (184.7–251)	<0.001
Effective orifice area (cm^2^), median (IQR)	1.7 (1.3–2.2)	1.5 (1.2–1.8)	2.0 (1.6–2.5)	<0.001
Effective orifice area index (cm^2^/m^2^), median (IQR)	11.0 (8.0–15.5)	0.8 (0.6–1.0)	1.1 (0.8–1.3)	<0.001
Effective orifice area index below 0.75 cm^2^/m^2^, *n* (%)	82 (34.7)	58 (49.6)	24 (20.2)	<0.001
Aortic valve velocity ratio, median (IQR)	0.5 (0.4–0.6)	0.5 (0.4–0.6)	0.6 (0.5–0.6)	0.01

Abbreviations: LVEF, left ventricular ejection fraction; SAVR, surgical aortic valve replacement; TAVR, transcatheter aortic valve replacement; TIA, transient ischemic attack.

**Figure 3:**
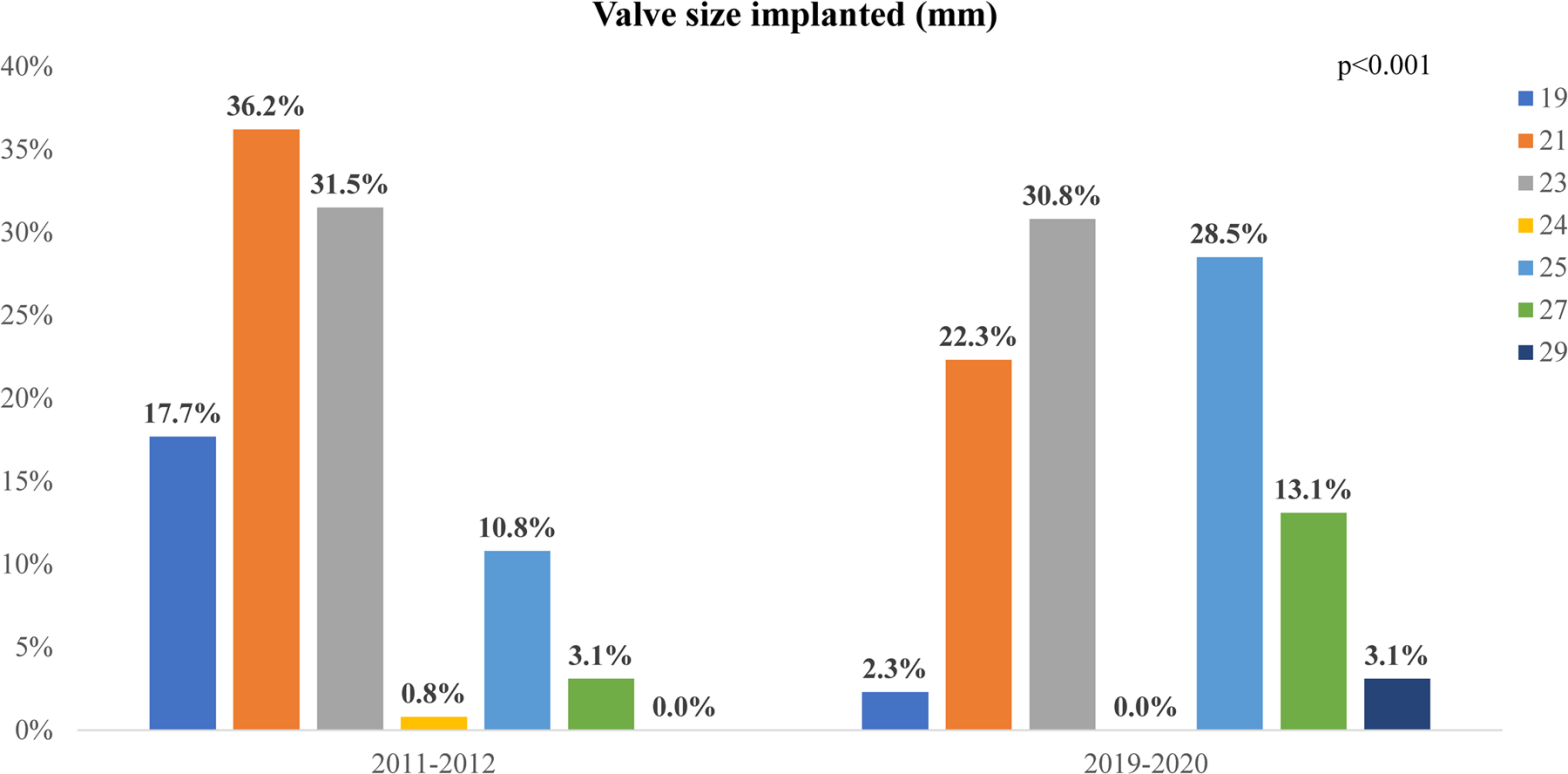
Distribution of valve size implanted in the study groups. A bar chart showing valve size distribution among patients who had a bioprosthetic SAVR procedure in 2011–2012 (early group, pre‐TAVR implementation) and 2019–2020 (contemporary group, post‐TAVR implementation). There is a significant difference in the distribution of valve size implanted across the years with a trend toward using smaller valves in the early group (*p* < 0.001).

The median valve size was significantly smaller in the early group as compared to the contemporary group (median of 21 (IQR: 21–23) vs. 23 mm (IQR: 22.5–25), *p* < 0.001). The distribution of valve size implanted across the years is shown in Figure 3, with a statistically significant difference in valve size between the groups. There was no difference in left ventricular ejection fraction after the surgery, 30‐day mortality, 1‐year mortality, the occurrence of cerebrovascular events, need for pacemaker implantation, need for TAVR, or need for dialysis across the groups (Table 2).

## 4 DISCUSSION

Our main finding is that in the contemporary era, surgical patients receive larger valves which translates into lower mean gradients, larger valve area, and lower rates of patient‐prosthesis mismatch than in previous years before the routine introduction of TAVR. Although there are likely multiple contributing factors as described below, this study stresses the importance of standardization of care to improve patient outcomes. The hemodynamic profile of TAVR has always surpassed that of SAVR valves.^1^, ^2^, ^5^ In patients with severe AS and increased surgical risk, TAVR has shown better survival at 1 year than SAVR.^6^ In patients who cannot undergo surgery, the PARTNER trial showed that TAVR carries a lower risk of morbidity and mortality than medical therapy.^1^ As for low and intermediate‐risk patients, TAVR has proven non‐inferior to SAVR in PARTNER 2 and other studies,^2^, ^7^, ^8^ and superior to SAVR in the PARTNER 3 trial in intermediate‐risk patients.^5^ In fact, a study done in our Veterans Affairs institution in Minneapolis in 2018 showed a decreased risk of stroke and mortality in TAVR vs SAVR.^9^ Notably, a study was done showing a 23.7% mortality at 1 year with TAVR but did not compare it to SAVR.^10^ In addition, Mack et al.^11^ showed in 2013 that in‐hospital death in the first 30 days following TAVR was mostly noncardiac.

Elevated mean gradients, whether in native or prosthetic valves, are associated with higher mortality and poorer outcomes.^12^ However, a study done in 2019 comparing SAVR and TAVR showed that TAVR is associated with less prosthesis–patient mismatch and lower transaortic gradients.^13^ There are likely several major reasons that contribute to an overall increase in gradients, lower valve area, and higher risk of patient‐prosthesis mismatch (PPM) in surgical patients. First, the engineering of a bioprosthetic surgical valve requires a suture ring which is used to attach the valve prosthesis to the native annulus. The valve itself must reside within the suture ring, which reduces the maximum valve area and increases the gradients across the prosthesis. This contrasts with the TAVR valves which are stented valves that lack a suture ring and can, therefore, accommodate a much larger valve area and attenuate the gradients across it. Second, implantation of a smaller valve is oftentimes surgically more feasible and technically easier and, as a result, more favorable despite the higher risk of associated PPM. Third, the overall patient profile of the contemporary versus the early group is younger and lower risk, which may translate into the surgeon's ability to implant larger valves. This trend is driven by TAVR approval in 2011 and its wider implementation in the contemporary era for inoperable, older, and overall higher‐risk patients.^1^ Fourth, routine use of contrast‐enhanced dedicated aortic valve computed tomography imaging may have led to a more accurate selection of aortic prostheses even in the surgical population. Surgical aortic valve sizing has always been primarily dependent on intra‐operative measurements using dedicated valve‐sizing balloons.^14^ The introduction of multidetector computed tomography in TAVR has revolutionized the sizing of TAVR valves and has been shown to be a dramatic improvement over the previous standard of care using two‐dimensional transesophageal echocardiography.^15^

The introduction of TAVR as a direct competitor to surgical valves showed that larger valve areas and lower mean gradients are possible with stented prostheses. This suggested that the overall TAVR profile is more favorable than the surgical valves. This was demonstrated in the PARTNER 1 and 2 clinical trials.^1^, ^2^ PARTNER 3, however, showed that hemodynamic profiles were much better for the SAVR cohort than in previous trials. This translated into much lower gradients and higher overall valve areas in the surgical cohort and seemed to bridge the gap previously seen between TAVR and SAVR. This suggests that the introduction of TAVR has led not only to the creation of a less invasive transcatheter approach but has also indirectly improved SAVR outcomes as well. The main purpose of this study was to independently investigate the outcomes of SAVR in the pre‐ and post‐TAVR era and determine whether the outcomes seen in PARTNER 3 are related to the Hawthorne effect or whether there has been a generational shift in SAVR toward implanting larger, more favorable prostheses.

Our study has a number of important contributing points. First, we demonstrate that the patients in the post‐TAVR era undergoing SAVR receive a valve with an augmented hemodynamic profile including lower mean gradients and larger valve areas. Second, we show that patients undergoing SAVR in the contemporary era have less patient–prosthesis mismatch. Third, patients in the contemporary group receive larger SAVR valves. Lastly, patients undergoing SAVR in the contemporary era have an overall better clinical characteristics profile and are at an overall lower risk than in the early years.

The debate over TAVR versus SAVR for younger and lower‐risk patients with severe AS remains unanswered. A study done in 2021 by Virgili et al.^4^ showed that SAVR is the go‐to for the younger population because they are typically better surgical candidates. In addition, a study by Mach et al.^16^ showed that although short‐term survival (30 days postoperative) is higher in younger patients undergoing TAVR, SAVR was associated with better long‐term survival. TAVR would be ideal for all ages given its less invasive nature and lower complication rate. In addition, if TAVR indications include low‐risk patients, it is expected to reach an annual candidate number of 270,000 annually in the USA and the European Union.^17^ However, several TAVR limitations remain, including the debate over valve durability, surgical challenges during future valve replacements, difficulty in accessing the coronaries, and most importantly the overall higher rate of pacemaker implants, which by itself trends with increased long‐term mortality and morbidity.^18^, ^19^

## 5 LIMITATIONS

The main limitation of our study is that it is a single‐center, nonrandomized, observational retrospective study. Nonetheless, complete data were obtained for our sample size and the results obtained are consistent with findings from previous studies. Second, the overall group size in both cohorts and the total cohort is overall small. Third, no propensity matching was performed due to the small sample size. Fourth, there was a lack of a core laboratory for echocardiographic measurements. Finally, no causality was found in this study which may limit the clinical applicability of our statistically significant results.

## 6 CONCLUSION

In the contemporary era, surgical patients receive larger valves which translates into lower mean gradients, larger valve area, and lower rates of PPM than in previous years before the routine introduction of TAVR. There are a number of unexplored endpoints related to this including increased attempts by surgeons to actively implant larger sizes, improved sizing tools such as routine computed tomography, and the overall more favorable surgical patient population. Further investigation is necessary to see what the broad impact of TAVR is on the surgical population and surgical technique, and how future structural procedures can indirectly favorably impact surgical outcomes.

## AUTHOR CONTRIBUTIONS

**Johnny Chahine**: conceptualization; data curation; formal analysis; investigation; methodology; software; writing—original draft; writing—review & editing. **Zeina Jedeon**: data curation; formal analysis; methodology; writing—original draft. **Jacob Fiocchi**: conceptualization; data curation; methodology; resources; software; writing—original draft. **Andrew Shaffer**: investigation; methodology; resources; supervision; validation; writing—review & editing. **Ryan Knoper**: conceptualization; investigation; methodology; resources; supervision; writing—review & editing. **Ranjit John**: conceptualization; investigation; methodology; supervision; validation; visualization; writing—review & editing. **Demetris Yannopoulos**: conceptualization; data curation; methodology; supervision; visualization; writing—review & editing. **Ganesh Raveendran**: conceptualization; funding acquisition; methodology; resources; software; supervision; validation; visualization; writing—review & editing. **Sergey Gurevich**: conceptualization; investigation; methodology; project administration; resources; software; supervision; validation; visualization; writing—review & editing.

## CONFLICTS OF INTEREST

The authors declare no conflicts of interest.

## TRANSPARENCY STATEMENT

All authors listed meet the authorship criteria according to the latest guidelines of the International Committee of Medical Journal Editors and are in agreement with the manuscript.

## Data Availability

The authors confirm that the data supporting the findings of this study are available within the article.
